# Managing disinformation on social media platforms

**DOI:** 10.1007/s12525-025-00796-6

**Published:** 2025-06-09

**Authors:** Eric K. Clemons, Maximilian Schreieck, Ravi V. Waran

**Affiliations:** 1 The Wharton School, University of Pennsylvania, 3730 Walnut Street, 572 Jon M. Huntsman Hall, Philadelphia, PA 19104 USA; 2 Department of Information Systems, Production and Logistics Management, University of Innsbruck, Universitätsstraße 15, 6020 Innsbruck, Austria; 3 Clearwater Paper Corporation, 601 W. Riverside, Suite 1100, Spokane, WA 99201 USA

**Keywords:** Fake news, Misinformation, Disinformation; Digital platforms, Regulation, D60, K24, L15, L50, L86, C63

## Abstract

Online disinformation campaigns are different from other attempts at deception that have existed for millennia, and they require new and more effective mechanisms to counteract them. Modern disinformation campaigns use individuals’ private personal information to craft stories carefully designed to manipulate specific readers. They again use this information to direct those stories to those readers who will be most sympathetic and away from readers who will detect the attempted manipulation and experience backlash. These campaigns undermine individual agency and human dignity and polarize societies, destroying societal cohesion, which is why disinformation campaigns have been called an existential threat to human civilization. Limiting the harm of disinformation is complicated by the need to preserve freedom of speech and avoid the appearance of bias, as well as by the resistance of social media platforms that find disinformation campaigns profitable. We use simulation modeling to explore the effectiveness of mechanisms to limit the spread of disinformation and offer guidance to regulators.

**Supplementary Information:**

The online version contains supplementary material available at 10.1007/s12525-025-00796-6.

## Introduction

Dis- and misinformation have been named the top global risk of the immediate term in the annual World Economic Forum (WEF) Global Risks Report 2025 (Elsner et al., 2025). We make the distinction between *misinformation*, or errors of any kind, and *disinformation*, which is the intentional crafting and distribution of stories that are known to be false and designed to manipulate public opinion (Aïmeur et al., 2023; Wardle & Derakhshan, 2017). In this paper, we focus solely on disinformation and, specifically, well-crafted campaigns that use online falsehoods to manipulate public opinion. The intention can be to manipulate an election and harm a political opponent, cause public health problems by interfering with a foreign power’s ability to get its population to accept vaccination, or for any other advantage.

Although attempts at manipulation have a history spanning millennia, online disinformation is more dangerous and more effective because it can be tailored to the interests and vulnerabilities of groups and individuals and directed to specific readers (Shao et al., 2017; Stöcker, 2020). Consequently, online disinformation is more effective than prior forms of propaganda and manipulation of public opinion, and it has become a threat to public health (George et al., 2021) to fair and open elections (Cadwalladr, 2017; Kaiser, 2019; Rapoza, 2017), to public safety (Silver & Frier, 2018), and even to societal cohesion (George et al., 2021). While traditional, brute-force propaganda, for example, in authoritarian regimes, can also be effective, it typically is accompanied by censorship and violent suppression of opposition—online disinformation campaigns can achieve manipulation in more subtle ways.

Disinformation has also been profitable for search engines and online social media platforms (Dizikes, 2018; Swisher, 2021; Vogelstein, 2018), which is one reason it has been challenging to get these platforms to limit the deliberate manipulation of their users (Cusumano et al., 2021). This is underlined by decisions by social media platforms to cut back on fact-checking programs (McMahon et al., 2025). Moreover, now that disinformation campaigns can be implemented with generative artificial intelligence (AI) tools, informed by massive online data sets, they are uniquely effective, so that companies and hostile foreign powers use them extensively (Cadwalladr, 2017; Levin, 2017; Rapoza, 2017; Rutledge, 2018; Kreps et al., 2022; Recorded Future, 2024). Online disinformation can cover attacks on political opponents’ reputations (Breiner, 2016), fake product reviews (He et al., 2022), or false descriptions of a politician’s platform, character, or personal life (Breiner, 2016; Clemons et al., 2024).

The problem of disinformation may appear intractable. Platforms are profiting from enabling disinformation (Dizikes, 2018; Swisher, 2021; Vogelstein, 2018) and have shown little incentive to modify their behavior or the behavior of agents that perform and benefit from disinformation campaigns (Owen & Silverman, 2022; Silver & Frier, 2018). Disinformation works, and agents that perform online disinformation campaigns have no incentive to modify their behavior, nor are they compelled to do so. Developing more restrictive regulations is difficult. One party’s disinformation is another party’s belief, and one party’s right to self-protection may appear to another party to be censorship and a violation of freedom of speech (Helmore, 2020; Thompson & Hsu, 2024).

However, the EU’s adoption of risk-based regulation and risk-based compliance (Efroni, 2021) may begin to alter the behavior of online platform operators. Social media platforms enabling disinformation campaigns may encounter stricter legal obligations to limit disinformation and more significant financial penalties if they fail. The EU has introduced the concept of risk-based regulation for novel and innovative applications. The most recent AI Act is the flagship for introducing this concept (European Union, 2024), but all online platforms are now required to take action to limit the risks they create for users and societies.

The information systems research has shown how digital technologies such as digital platforms and generative AI foster the crafting and targeting of disinformation (e.g., Feuerriegel et al., 2024). Further studies have analyzed how disinformation spreads (e.g., Moravec et al., 2018; Shao et al., 2017), how disinformation can be identified (e.g., Wei et al., 2022; Zhang et al., 2019), and have suggested countermeasures such as flagging content (e.g., Kim et al., 2019; Moravec et al., 2020) or pre-bunking (e.g., Ecker et al., 2014). However, we lack insights into how different interventions impact the spread of disinformation and how this can be evaluated systematically. We therefore post the following research question: *To what extent can restrictions on sharing user data and forwarding content limit the spread of disinformation on social media platforms?*

To address this research question, we implement a simulation model (see also Clemons, 2018). We use simulation for two reasons: First, it allows us to create a dynamic model and explore trajectories over time. Closed-form equilibrium models can tell us that the equilibrium is that the disinformation campaign succeeds or fails. A simulation model allows us to view behavior over time. It allows us to determine that while a disinformation campaign might eventually succeed with certain assumptions about parameter values, it would not succeed in time to influence an upcoming election. Second, it allows us to see how changes in parameter values, either individually or in conjunction with other changes, alter the trajectory. For example, we can see how changing assumptions about how often stories get reposted interact with the limitations or delays that need to be imposed on the most egregious reposting to alter the success or failure of a disinformation campaign.

We show that increased information sharing between the platform and the operators of disinformation campaigns improves the campaign’s effectiveness; this has been documented by Wylie (2019) and others. We show how this information sharing benefits the platform by improving both the targeting and the precision crafting of stories, enabling shorter campaigns with reduced opportunities for backlash. We show how even apparently safe, aggregate information sharing can enable successful disinformation campaigns. Moreover, we show how slowing the reposting of the most egregious fake news stories can reduce the effectiveness of disinformation campaigns without infringing on the original posters’ freedom of speech.

With these findings, we contribute to the information systems literature on disinformation by first showing the effectiveness of targeted disinformation campaigns. Even when social media platforms do not share individual-level user data with third parties, their ability to serve content to sympathetic readers makes disinformation much more effective than broadcast disinformation. Second, we evaluate several countermeasures and find that restricting forwarding can be a promising mechanism to limit the spread of disinformation. We thereby add to the growing stream of literature on combating disinformation. We further contribute to practice by highlighting implications for regulators, platform owners, and society.

## Background—Prior research on disinformation

In this section, we will provide background on the rising issue of online disinformation and consider the EU’s risk-based regulation in the context of disinformation. This overview highlights the need to find more effective ways to manage disinformation.

### The challenge of disinformation

Online misinformation and disinformation are a growing problem (Kim & Dennis, 2019; Kim et al., 2019; Moravec et al., 2018). While misinformation refers to any wrong information, disinformation requires the intentional distribution of incorrect information to cause harm (Aïmeur et al., 2023; Wardle & Derakhshan, 2017). Therefore, the term fake news often refers to disinformation (Kim & Dennis, 2019; Kim et al., 2019). Our paper focuses on disinformation campaigns, orchestrated approaches to distributing a portfolio of wrong information—or fake news stories—to manipulate people or cause other forms of harm. Thereby, not all information in the disinformation campaign has to be false; often, the campaigns deliberately combine true and false information in complex ways to increase legitimacy and deceive readers (Chadwick & Stanyer, 2022).^1^

Disinformation can be effective because, on social media platforms, false information spreads faster than true information (Vosoughi et al., 2018). Explanations for this phenomenon put forth by Vosoughi et al. (2018) include that false information is perceived as more novel than true information and triggers emotions such as surprise, disgust, and fear, all leading to more forwarding of the information. Disinformation campaigns can be even more effective if they can be targeted to a specific audience based on information about the individuals in the target audience (Shao et al., 2017; Stöcker, 2020). Notorious disinformation campaigns include one designed to convince British citizens to vote for Brexit by distributing false claims about the cost of EU membership, the loss of employment, and the risks to the British National Health Service (Cadwalladr, 2017; Kaiser, 2019). During the COVID pandemic, disinformation campaigns spread conspiracy theories about masking and vaccinations (Frenkel et al., 2020; van der Linden et al., 2021), increasing the severity of the pandemic in the USA.

Previous research has found evidence of the harmful impact of disinformation. For example, it has been shown that the increasing spread of disinformation can accelerate the polarization of our societies (Casal Bértoa & Rama, 2021; French et al., 2024; Qureshi et al., 2022). Furthermore, there is evidence that foreign governments engage in disinformation campaigns to influence public opinion, for example, before elections. Beskow and Carley (2020) lay out indications for Chinese and Russian disinformation campaigns. For instance, whether or not Russian disinformation actions affected the 2016 US Presidential Elections, Moscow had a strong preference for Trump over Clinton (Rapoza, 2017).

The threat created by disinformation is becoming more urgent as generative AI tools reduce the cost of creating disinformation and increase its effectiveness (Jaidka et al., 2024; Shin et al., 2024). Generative AI tools can be used to produce well-crafted and well-targeted disinformation at scale and high speed (Kreps et al., 2022), and they make it easier to personalize disinformation to target audiences and individuals (Feuerriegel et al., 2024). Generative AI can also create visual and audio material, including pictures and videos (also called deepfakes), that become increasingly indistinguishable from accurate material (Vaccari & Chadwick, 2020). This material not only makes disinformation campaigns more effective but also reduces overall trust in the news and media (Vaccari & Chadwick, 2020). First, studies and reports indicate that deepfakes play a role in election campaigns (Łabuz & Nehring, 2024) and that generative AI has been used in campaigns related to the 2024 US presidential election (Recorded Future, 2024). Specifically, generative AI was used to generate inauthentic news websites with fake journalist personas to publish disinformation disguised as legitimate news articles (Recorded Future, 2024). In the future, the automated generation of text at scale combined with fake audio, photo, and video material indistinguishable from true material will further fuel the spread of disinformation (Elsner et al., 2025).

Consequently, we see more and more calls to limit disinformation online. For example, Nobel Peace Prize laureate of 2021, Maria Ressa, criticizes the big tech platforms for letting disinformation spread uncontrollably, pointing to disinformation campaigns during elections in the Philippines (Ressa, 2021, 2022). Renowned tech journalist Kara Swisher highlights the role of disinformation campaigns around the January 6 th pro-Trump riots in Washington and calls out social media platforms like Facebook and X (formerly Twitter) for not limiting the spread of disinformation (Swisher, 2020, 2021). However, the companies behind the largest search engines and social media platforms benefit from disinformation campaigns, given that they often increase engagement on the platforms (Dizikes, 2018; Swisher, 2021; Vogelstein, 2018). Thus, they have not been consequential in combating disinformation and have begun cutting back fact-checking programs, at least in the USA (McMahon et al., 2025).

Prior literature has found that it is challenging to limit disinformation. Several approaches have been proposed to identify disinformation, for example, relying on machine learning techniques and further analytics approaches (Shu et al., 2020; Zhang et al., 2019) by combining machine learning and crowd feedback (Wei et al., 2022) and by analyzing the credibility of the authors (Sitaula et al., 2020). While identifying disinformation is already challenging, limiting its spread is an even more daunting task. First, several studies have evaluated mechanisms that label or flag disinformation content. However, findings are mixed, and labeling alone is insufficient to significantly limit the spread of disinformation (e.g., Kim et al., 2019; Moravec et al., 2020). Second, other suggested approaches include debunking and pre-bunking of disinformation. While debunking relates to countering disinformation with true information, pre-bunking—or inoculation—refers to educating consumers to help them identify disinformation (Ecker et al., 2014; Roozenbeek et al., 2020). However, these efforts have been shown to be of limited effectiveness because of the consumers’ confirmation bias. That is, readers tend to believe information that fits their beliefs, opinions, and worldviews (Moravec et al., 2018), and scientific and well-managed disinformation campaigns use private information to craft and direct stories that fit the beliefs, opinions, and worldviews of their recipients.

Third, the role of fact-checkers has been analyzed, and recent studies suggest that fact-checking could be enhanced by automated identification of disinformation and generative AI tools to prepare responses to disinformation posts (Vo & Lee, 2020). To the best of our knowledge, the impact of such measures has not yet been evaluated at a large scale. Fourth, limiting the visibility of disinformation content on platforms as an alternative to removing content has been suggested, and it has been documented that social media platforms employ the technique (Gillespie, 2022). However, the effectiveness has not been evaluated yet. Lastly, there have been calls for social media platforms to self-regulate, but even when platforms signaled commitment, there was little evidence that this was effective. For example, YouTube self-committed to limit disinformation videos in the context of the Brazilian General election, but evidence was found that disinformation content could still be spread easily (Santini et al., 2023).

As a result, restricting the spread of disinformation is also a daunting challenge for regulators. There is a complicated balance between ensuring freedom of speech and combating the harm of disinformation (Helmore, 2020; Nuñez, 2019; Thompson & Hsu, 2024). If government agencies or other private actors were to decide what content on social media platforms is true and false, this could easily be abused by political actors to limit content they consider unfavorable to them and their policies. Additionally, lobbyists from parties with vested interests, including political parties and companies that may benefit from disinformation, pressure regulators to limit regulatory efforts.

### The EU’s risk-based regulation in the context of disinformation

A regulatory approach to combatting misinformation is risk-based regulation, a strategy that is gaining traction in the regulation of information technology (Butler et al., 2023; Ullrich, 2018). Risk-based regulation starts with the idea that regulators do not know enough to specify all risks that innovative online platforms may create and thus cannot know enough to list all behaviors that should now be prohibited. Companies must take action to preemptively limit the harm that their platforms and systems create, even when regulators have not explicitly suggested which actions must be restricted or prohibited, and even when companies may not know how their platforms will be used or how they will interact with other systems in place at some future time. The innovator is then required to manage all future forms of harm. This creates potentially open-ended liability since the presence of harm can be seen as a concrete and irrefutable failure of compliance with risk-based regulation, with damages assessed even though the platform violated no explicit regulations. The mere presence of future harm can be interpreted as a compliance failure.

The EU’s intentions for risk-based regulation and risk-based compliance can be found in the requirements provided in the AI Act. As a striking but not unrealistic example, suppose a hostile foreign power were to use an online platform to create significant resistance to routine childhood vaccinations, and suppose that the platform operator did nothing to limit the disinformation campaign despite scientific evidence demonstrating the safety of most childhood vaccinations. Suppose, moreover, that a major outbreak of measles, polio, or whooping cough were to strike the EU as a result of a drop in childhood vaccinations. If causality could be established, consider the magnitude of the platform operator’s liability.

Companies are not required to perform traditional risk management in the sense that a software engineer might consider the term. In these cases, the platforms operate precisely as they were designed to manage and perform strictly as intended. Moreover, the platforms violated no laws or regulatory restrictions during the execution of the disinformation campaign. Here, companies are required to avoid the risk of unintended consequences. This requires an entirely different mindset from software engineers and a behavior change from corporate executives and corporate boards of directors.

After decades of study, reducing the risk of software failures is relatively well understood. We have less understanding of the risk of unintended consequences and the harm caused by systems that execute strictly as intended but produce harmful and unanticipated side effects. A historical example—portfolio insurance—can illustrate the complexity of understanding the interactions among a platform, its users, its future operating environment, and other actors. While not related to disinformation, this example shows the extent of liability that the incomplete specification of risk-based regulation could create.

Portfolio insurance was designed in the mid-1980s by the largest investment management firms to protect their clients from future declines in the value of their investments (Jacklin et al., 1992). The systems executed strictly as intended but failed to protect the firms’ investments. Indeed, the impact of portfolio insurance was quite the opposite of what was intended. This form of computer-initiated program trading led to the dramatic market crash of 1987, in which the market crashed with unprecedented speed (Jacklin et al., 1992; Shiller, 1988). The idea behind portfolio insurance was that if the market started to decline and investors were beginning to lose money on the shares they owned, they could make bets in the futures market that shares would continue to fall. With these winning bets in the futures market, they could earn enough to offset their losses in the stock market. This would have worked if the system had been used by one investor or perhaps even by investors in a single firm. What happened was that the widespread simultaneous selling of futures by a vast number of investors sent the signal that people were betting against the stock market, which caused share prices to decline further. This was a positive feedback loop, in which the more the market dropped, the more portfolio insurance caused it to drop further. This led to a general panic and an unprecedented market crash. Portfolio insurance, in the end, protected no one and contributed instead to a global recession.

Most significantly, for the creators of future innovative online platforms, this example suggests the possibility that their platforms will create risks they did not anticipate and harm they did not prevent. The presence of this harm may be considered sufficient evidence for a failure of risk-based compliance. This suggests that the operators of social media platforms will need a profound understanding of the risks their platforms may create, especially relative to their enabling of disinformation campaigns. Below, we present an analysis of how to limit the impact of disinformation campaigns, which may be essential to limiting the future liability of social media platform operators.

## Methodology—A simple model of disinformation campaigns

We developed a simulation model of the factors that affect the speed of propagation of disinformation, the effectiveness of disinformation campaigns, and interventions that limit the spread of disinformation (the model is presented in more detail in Appendix A).^2^ We constructed the simulation model in GoldSim (2022). With the simulation, we are not seeking equilibrium analysis, but rather the behavior of complex human systems over time, for which simulation is most appropriate (López-Paredes et al., 2012). Simulation has a long and respected history in economics (Meier et al., 1969; Ruth & Hannon, 2012) and in the other social sciences (Hartmann, 1996; Squazzoni et al., 2014), especially when it is necessary to explore dynamic behavior, and when it is needed to explore structural models and examine the impacts of changing various parameters on the behavior of the system. Below, we will outline the model’s assumptions.

In simulation modeling, as with all modeling efforts, initial assumptions about parameter values and the strength of interactions will alter the outcomes of the simulations and the findings that the simulation produces. With closed-form mathematical models, the parameters and their values are essential to the model’s presentation. In contrast, with simulation modeling, many of the parameter values and even the role of the parameters themselves are hidden within the model’s code. Our parameters and their values are listed in the discussions of the model and its findings. However, we have prepared a table that lists all of the parameters used in the model and the values we assumed for them (Appendix B). We describe how changes in these values would alter the model’s findings, and in many cases, we performed and documented sensitivity analyses. The table is presented in an appendix. The detailed description of the model is also presented in an appendix.

We model a referendum where Green opposes Red, where Green initially enjoys a slight electoral majority, but Red engages in one of several disinformation campaign “treatments.” Assume any pair of Red and Green populations representing opposing views. We do not assume that either Red or Green is correct. We assume that Green initially enjoys greater voter support and that Red employs a carefully constructed disinformation campaign to reverse this and gain a voting majority.

We make the following assumptions about the *initial distribution of voters* (Table 1). (1) Potential voters are distributed non-uniformly over a 2-dimensional voter-attitude space. (2) Voters are distributed in segments (columns in the table) from left to right, with extreme Red believers at one end and extreme Green believers at the other. (3) Red is represented by Segment 2, and Green by Segment 7. Thus, there are more extreme Red views (column 1) and Green views (columns 8 and 9) than the positions of Red and Green in the referendum. This is in line with elections where candidates often are not aligned with the most extreme views of voters. As a result, the most extreme voters might not actually vote for the position or candidate closest to them (Jones et al., 2022). (4) Voters are also distributed in levels or rows in the table, from “bottom” to “top,” with the most committed at the top and the least committed at the bottom.

**Table 1:**
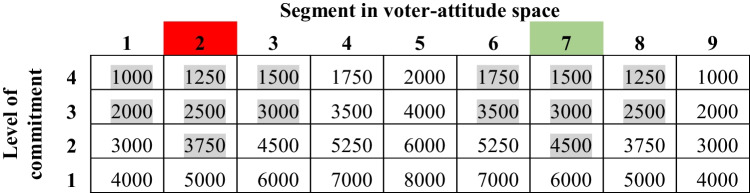
Distribution of voters in voter-attitude space

Our model of *voter behavior* assumes that voting is a function of both belief segment and commitment level: (1) The top three levels of segment 2 and the top two levels of adjacent segments 1 and 3 vote for Red (grey cells). Likewise, the top three levels of segment 7 and the top two levels of segments 6 and 8 vote Green. These populations are highlighted in Table 1; the other potential voters in the voter-attitude space do not vote. Two things are important to note here. First, voters in segment 9 do not vote. As shown in segment 7, Green’s position is not extreme enough to motivate voters to vote (Jones et al., 2022). For example, in the most recent US presidential election, many progressive democrats chose not to vote because they believed that Kamala Harris did not represent their views; as a result, they ended up with a government that was even less responsive to their beliefs (Bowden, 2024). Second, we have located Red in a more extreme segment than Green. We did this for two reasons. First, it is now known that disinformation campaigns are frequently employed by the more extreme of the alternatives (Bradshaw et al., 2020). Second, we needed Red to start with a lower voting population to see if disinformation altered the outcome of an election in Red’s favor, and the simplest way to treat this was to let the relative demographics of segments 2 and 7 provide the imbalance we required.

We make the following *behavioral assumptions about the impact* of individual disinformation stories. (1) Stories that are well-targeted are aimed at the segment that fully endorses the argument advanced by the disinformation and at the two segments adjacent to it on either side. (2) An individual well-targeted story can move a specified percentage of voters one segment closer to the target (16%), one segment more committed (12%), or both (9%). (3) Stories that are not targeted are seen by all readers, including readers whose views are unsympathetic to those advanced by the story. When stories are seen by unsympathetic readers, we call this *leakage*, and leakage can produce the opposite of the intended response, which we call *backlash*. Backlash can result from unsympathetic readers becoming more fervent supporters of their political cause or more committed to voting. A backlash could also be an indirect effect, for example, of grassroots initiatives combating disinformation (Weir, 2024). (4) Backlash has a smaller impact than the campaign’s impact on its target. It can move a specified percentage of voters one segment closer to the target’s opposition (12%), one segment more committed (8%), or both (6%). (5) Backlash to Red’s disinformation affects only segments 5, 6, and 7, and only if they are exposed to the disinformation campaign. We assume segments 8 and 9 are already more extreme than 7 and do not move closer to Green due to disinformation; we believe this is more conservative than allowing Green to obtain new voters from more segments from backlash than Red attracts with its disinformation campaign.

We perform sensitivity analyses to assess when our model would offer different predictions with different parameter values (see Table 2). (1) We vary the relative advantage of disinformation’s impact on its targeted readers relative to the backlash created by leakage. (2) We vary the precision crafting of disinformation, which allows shorter campaigns with a lower possibility of leakage and backlash. (3) We vary the starting time of backlash, allowing it to occur sooner or later and allowing it not to occur at all for short enough precision-targeted campaigns. (4) We vary the sensitivity of different groups to detecting disinformation manipulation by varying the time during a campaign before different groups begin to experience backlash (“ramp-up” of backlash).

**Table 2:** Summary of validity treatments

Treatment name	Validity 1		Validity 2		Validity 3		Validity 4
**Description**	Broadcast, untargeted disinformation		Narrow-cast, targeted disinformation		Narrow-cast, targeted disinformation with variation in backlash propensity		Narrow-cast, precision-targeted disinformation
**Analysis type**	Main	Sensitivity	Main	Sensitivity	Main	Sensitivity	Main
**Parameters**							
Backlash starts	0	0	8	8	2	5	8
Backlash intensity	0.75	0.25	0.75	0.65	0.75	0.75	0.75
Backlash ramp-up length	-	-	-	-	10	20	-
Red precision increase	0	0	0	0	0	0	0.2
Campaign length	15	15	15	15	15	15	8
**Outcome**							
Red victory-period	-	12	-	14	-	10	8
Red victory-percentage	-	1.18	-	0.53	-	2.16	0.16

## Simulation results

Before using a simulation model to perform experiments, we test the model’s face validity (Klügl, 2008) and assess whether or not the behavior of the model is plausibly correct. We ask, do long disinformation campaigns impact the intended target population more than very short ones? If the disinformation campaign is long enough, does backlash become more significant? Next, we perform a sensitivity analysis (Kleijnen, 2009) to demonstrate that the model is stable and that small changes in parameter values do not have unreasonably large impacts on the model’s behavior. If the model behaves as expected under a wide range of conditions and changes in parameter values, we have increased confidence in the model’s legitimacy. There is no such thing as proof by exploring a complete set of examples or proof by exhaustion, and the failure to find a counter-example is not proof of the absence of a counter-example (Klügl, 2008), but this is a good way to increase our confidence in the model (Kleijnen, 2009).

### Model testing

We use the following four Face Validity Treatments in the first stages of testing our model:

- *Broadcast, untargeted disinformation*, in which the same stories are sent to all users, to explore the impact of traditional propaganda campaigns.
- *Narrow-cast, targeted disinformation*, in which stories are carefully sent to likely sympathetic users. However, when campaigns are too long, leakage occurs, unsympathetic users view the stories, and backlash can occur.
- *Narrow-cast, targeted disinformation with variation in backlash propensity*, in which we explore how sensitive the model’s results are to backlash.
- *Narrow-cast, precision-targeted disinformation*, in which stories are carefully crafted to appeal to small groups and carefully targeted to only those groups. Since stories are more carefully crafted and more precisely targeted, disinformation campaigns can be shorter, and the impacts of backlash can be reduced.

**Fig. 1:**
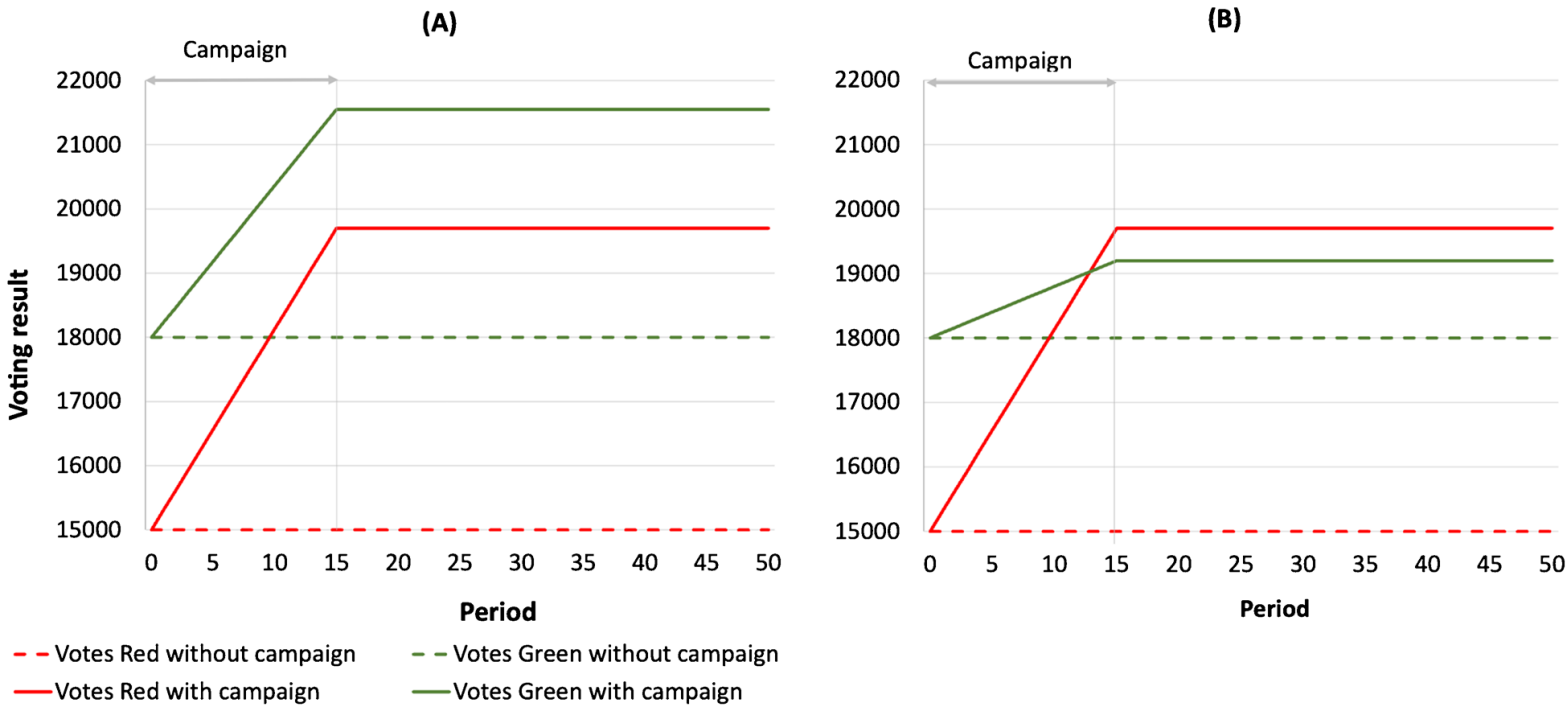
**A** Broadcast, untargeted disinformation campaign with high backlash intensity. **B** Broadcast, untargeted disinformation campaign with low backlash intensity

Figure 1 provides an example of the results of a pair of simulation runs. Figure 1A corresponds to column 1 in Table 2, with untargeted disinformation and high backlash intensity, Green remains victorious. With much lower backlash intensity, the disinformation campaign’s backlash does not motivate Green to the same extent, and Red’s campaign is successful.

Columns 1 and 2 explore the impact of disinformation campaigns created without access to users’ personal information to carefully craft stories for individual populations and without the information required to target stories only to sympathetic readers. Here, stories are broadcast and sent to all individuals. Column 1 shows that the effect of backlash when a long campaign alienates Green supporters can be sufficient to keep the disinformation campaign from succeeding. Column 2 shows that if the impact of backlash on Green supporters is low relative to the impact of stories on Red supporters, then even untargeted disinformation campaigns can succeed.

Columns 3 and 4 explore the effect of narrow-casting, of targeting stories only to individuals sympathetic to Red. Backlash still occurs, but only after 8 periods. Targeting delays leakage, and disinformation will initially spread within “Red” filter bubbles (Arguedas et al., 2022), but eventually, individuals sympathetic to Green will become aware of the disinformation campaign and experience backlash. Backlash is still sufficient to prevent Red from defeating Green, but even a slight reduction in Green’s sensitivity to disinformation is enough to enable Red’s victory.

Columns 5 and 6 assume that different customers have different sensitivity to disinformation and different propensity to experience backlash; thus, backlash ramps up over time. In column 5, backlash starts in period 2 with only 1/8 of the population susceptible, and the entire population is not susceptible until period 10; this slow ramping up of backlash begins early enough to preserve Green’s advantage. In column 6, backlash does not start until period 5 and is not entirely ramped up until period 20; in this case, the backlash is insufficient to neutralize Red’s disinformation campaign.

Column 7 explores the narrow-casting of precision-crafted stories. The platform shares enough personal information on its users with the disinformation campaign to enable the disinformation campaign to craft different stories for specific groups of individuals. This crafting ensures that stories are effective and resonate with the readers. Precision crafting and precision targeting allow campaigns to be more effective and much shorter, significantly reducing opportunities for leakage to Green supporters and greatly reducing the backlash that leakage produces. This combination, using data to precision craft stories and precision target them, is sufficient to ensure an early Red victory. Column 7 is the most interesting, and we return to this case in more detail in the section on First Interventions—Restricting Platforms’ Sharing of User Data.

In every case, in every treatment, and for every parameter manipulation, the change in treatments and parameter values produced the expected result. These tests of face validity suggest that the duration of a disinformation campaign, the design of specific disinformation messages, and the accuracy of targeting disinformation messages all contribute to the effectiveness of a disinformation campaign.

### First interventions—Restricting platforms’ sharing of user data

We next explore whether regulators can reduce the effectiveness of disinformation campaigns by limiting platforms’ sharing data on their users with the groups or individuals responsible for creating and sharing disinformation. Here, we assume that the platform operators rigorously adhere to EU privacy requirements and share no individual’s data with any outside parties attempting to implement a disinformation campaign. We presume that platform operators can still share aggregate data on entire populations. We also assume that platform operators are still allowed to use individuals’ data internally to send them material that they will find engaging and offer them a superior experience since this is consistent with EU privacy policies.^3^ Our results show that, at present, a platform operator can fully comply with EU privacy policies regarding paid content while enabling disinformation campaigns to execute efficiently. It remains to be seen how future courts will assess platform operators’ complicit responsibility for harms caused by these disinformation campaigns.

The combination of sharing aggregate data or not and targeting stories or not leads to four distinct treatments, all consistent with the EU’s privacy restrictions. Each implies a different degree and type of cooperation between the platform operator and the disinformation campaign. Thus, each implies a different level of complicity and future legal liability for the platform operator under risk-based regulation.

- *No data is shared with disinformation campaigns, not even aggregate user data, and the platform does not facilitate targeting of disinformation*. This corresponds to column 1 in Table 3. Even if long-running, disinformation campaigns are ineffective with little impact on Red and Green votes. Platform operators should not anticipate litigation or vulnerability under risk-based regulation and risk-based compliance.
- *The platform shares aggregate data on the interests, concerns, and vulnerabilities of different groups of users, but the platform does not facilitate targeting of disinformation*. This corresponds to column 2 in Table 3. Stories are more effective since they are crafted with information on user populations and attract less backlash (i.e., less impact on Green votes than for the previous treatment). However, without precision targeting, disinformation campaigns are still not effective, and platform operators should not anticipate litigation or vulnerability under risk-based regulation and risk-based compliance.
- *No data is shared with disinformation campaigns, not even aggregate user data, but the platform targets disinformation at interested users*. This corresponds to column 3 in Table 3. Disinformation stories are targeted using aggregate user data. Thus, they are not precision-targeted because information on the interests, concerns, and vulnerabilities of different groups of users is not available. Still, targeting based on aggregate user data significantly impacted Red votes, even in shorter campaigns. The overall impact of the disinformation campaign is uncertain because the intensity of the backlash against visible disinformation campaigns strongly influences it. Platform operators may wish to protect themselves by assessing the impact disinformation has on sets of individuals. Platforms know which users are and are not vulnerable and could conduct small surveys to ascertain how effective disinformation is. This would enable them to assess their future liability.
- *The platform shares aggregate data on the interests, concerns, and vulnerabilities of different groups of users, and the platform targets disinformation at interested users*. Stories are crafted to appeal to small segments of users, and the platform knows enough to target individual stories to the users who will find each story most engaging (column 4 in Table 3). This combination is sufficient to reverse the outcome of an election and give the victory to Red because the backlash is reduced compared to the previous treatment (i.e., a smaller impact on Green votes). This type of campaign is feasible even without the platform sharing any individual’s private data and without the platform violating privacy regulations. Aggregate data is shown to be sufficient for crafting stories with high impact, as long as the platform knows enough for precision targeting, and the platform’s precision targeting ensures impact that a short campaign can be sufficient. These short campaigns create only limited possibilities for a backlash among Green voters.

**Table 3:** Summary of data sharing treatments

Treatment name	Data sharing Treatment 1	Data sharing Treatment 2	Data sharing Treatment 3	Data sharing Treatment 4
**Description**	No user data shared by platform; no targeting	User data shared by platform; no targeting	No user data shared by platform; with targeting	User data shared by platform; with targeting
**Parameters**				
Backlash starts	0	0	0	0
Backlash intensity	0.75	0.75	0.75	0.75
Red precision increase	0	0	0	0
Campaign length	75	75	15	15
Impact on red votes	0.15	0.15	1	1
Impact on green votes	0.075	0.05	0.5	0.4
**Outcome**				
Red victory-period	-	-	-	14
Red victory-percentage	-	-	-	0.57

The fourth of these conditions demonstrates that enforcing privacy regulations is insufficient to eliminate the danger of disinformation campaigns. Likewise, a website or platform that fully complies with EU privacy regulations can still contribute to successful, harmful disinformation campaigns.

Exploration of the fourth set of conditions shows that platform operators who cooperate fully with disinformation campaigns enable those campaigns by greatly increasing their effectiveness. Under risk-based regulation, platform operators can be held responsible for the campaigns they enabled and for the harm they caused, even if they did not violate any explicit regulation concerning privacy or the dissemination of information.

### Second intervention—Imposing restrictions on forwarding content

We now consider a more complex set of trajectories for stories involving posting and forwarding. We now assume that the campaign notifies a small number of influential individuals (*Influencers*) and gives each influencer a list of sympathetic readers susceptible to their influence (Goodwin et al., 2023). The disinformation campaign is assumed to have access to personal information that helps target stories to the right group of susceptibles for each story. This information is now widely available to political campaigns and can readily be inferred from publicly visible data on users’ social media home pages. Additionally, we assume that stories forwarded from a known influencer have more impact than a story encountered by another means.

Our expanded model now has four new parameters.

- *Influencers*: This is the number of influencers who receive the story directly without reposting
- *Influencer impact*: This is the relative impact of a story from a known contact rather than from another source that is not trusted. Influencers have persuasive power over consumers (Liu & Zheng, 2024), and we assume that this also translates to the political sphere. This affects the extent to which the story alters a recipient’s beliefs and the likelihood that the recipient will forward the story to additional users in their network.
- *Forwards*; This is the number of times each influencer forwards the story to known susceptibles. Forwards are the essential element of a story “going viral.” We assume that forwarding occurs at a significant level because it is assisted in some way by the social media platform.
- *Waves*: This indicates the number of waves of forwards we assume. A campaign can achieve 1000 forwards either as a single wave of 1000 or as three waves of 10, 100, and 1000.

The most important change made in the extended model is to consider the impact of allowing users to repost existing content and examine what happens when a story goes viral. We also explore what happens when restrictions are implemented that limit some stories from going viral.

- *Forwarding Treatment 0, validation*: As shown in column 1 in Table 4, the model produces the same results as the initial model when parameter settings are equivalent. This is important to establish that the new simulation produces the same results as the previous one under the same conditions. This increases our confidence that changes in the output result from changes in how the model treats the newly added conditions and that they are not due to unintended changes in the model.
- *Forwarding Treatment 1, reposting in waves, no restriction on user behavior*: As shown in column 2 in Table 4, when a story goes viral, it has sufficient impact to change the outcome and give Red a victory.
- *Forwarding Treatment 2, reposting in waves, restricted initial posts*: As shown in column 3 in Table 4, when stories achieve their impact through waves of forwards, restricting Red’s most egregious initial posts is sufficient to preserve Green’s victory. However, restricting initial posts may be considered censorship. Moreover, additional simulations (not shown) demonstrate that if the disinformation campaign dramatically increases the number of initial posts, this may be sufficient to counter the restriction on reposting, and a hostile state actor almost certainly has the resources to overwhelm this attempted intervention.
- *Forwarding Treatment 3, reposting in waves, restricted reposting of suspect stories*: This is shown in column 4 in Table 4, and it is the intervention that seems most promising. The platform operator must now restrict the forwarding and reposting of the most suspect stories. The assumption is that each of these stories can be assessed objectively and algorithmically, reducing the danger of platform operators exhibiting bias and reducing the likelihood that they are accused of bias.

**Table 4:** Summary of forwarding treatments

Treatment name	Forwarding Treatment 0	Forwarding Treatment 1	Forwarding Treatment 2	Forwarding Treatment 3
**Description**	Validation	Reposting in waves; no restriction on user behavior	Reposting in waves; restricted initial posts	Reposting in waves; restricted reposting of suspect stories
**Parameters**				
Backlash starts	8	8	8	8
Backlash intensity	0.75	0.75	0.75	0.75
Red precision increase	0.25	0.25	0.25	0.25
Campaign length	8	8	8	36
Influencers	50	50	50	100
Influencer impact	4	4	4	4
Forwards	1,000	10	9	6
Waves	1	3	3	3
**Outcome**				
Red victory-period	8	8	-	-
Red victory-percentage	0.19	0.20	-	-

In Treatment 3, we showed that if forwarding of suspect content is restricted, then the impact of disinformation campaigns can be limited, even if the campaign runs longer and with more influencers. Social media platforms are unlikely to implement these restrictions without protest since the most egregious stories often generate the greatest user engagement and profits. However, since these restrictions do not involve limiting an individual’s right to create content, they may be less likely to be criticized as censorship and may be successfully imposed by regulators. The paper by Herb Lin in this special issue addresses mechanisms for assessing which stories are most reliable and which are most suspect (Lin, 2024).

## Discussion

Our simulation study has shown, first, that disinformation campaigns are more effective the better they can be crafted for specific, sympathetic audiences and the better they can be targeted toward this audience, avoiding backlash from unsympathetic audiences. For effective disinformation campaigns, drawing on aggregate user data to craft stories and the platform’s mechanisms to target stories to receptive audiences can be sufficient. Thus, even when platform operators adhere to the EU’s privacy and data protection regulations, they can effectively be enablers of disinformation campaigns. Second, assuming that removing disinformation is challenging, we show that limiting the spread by reducing forwarding can effectively combat disinformation. These findings have implications both for the current literature on disinformation and for practice.

### Contributions to literature

Our contribution to the literature on disinformation is twofold. First, we contribute to our understanding of the effectiveness of disinformation campaigns (cf. Kim & Dennis, 2019; Kim et al., 2019; Moravec et al., 2018). It has already been shown that false information spreads faster than true information (Vosoughi et al., 2018) and that disinformation campaigns can be effective by relying on social bots, targeting specific audiences, and by being amplified by the platforms due to their high level of engagement (Shao et al., 2017; Stöcker, 2020). We add to this literature stream by showing that using the aggregate user data available for third parties from social media platforms can significantly enhance disinformation campaigns’ effectiveness, particularly when platforms serve content to receptive audiences. This happens automatically as content is displayed in users’ feeds based on the platforms’ algorithms and can even be enhanced through paid campaigns. Only users opting out of targeted advertisements would not be receptive to such campaigns. These users, however, would still see posts in their feed, for example, based on the accounts they follow. In sum, sophisticated disinformation campaigns can employ combined approaches, using social bots, influencers, and paid campaigns to spread their stories. Future research could study the interplay and synergies of these approaches.

Second, we add to the current research effort on countermeasures for disinformation. Previous research has found little evidence that flagging content is sufficient to combat disinformation (e.g., Kim et al., 2019; Moravec et al., 2020). Similarly, pre-bunking and fact-checking do not appear promising (Ecker et al., 2014; Roozenbeek et al., 2020). Pre-bunking struggles to overcome the consumers’ confirmation bias (Moravec et al., 2018), and fact-checking struggles to keep up the pace in the face of ever-increasing amounts of disinformation. Our simulation shows that restricting forwarding can be an effective way to limit the spread of disinformation. This aligns with previous work that provides anecdotal evidence for such content moderation measures (Gillespie, 2022). Future research could combine approaches to identifying disinformation (e.g., Shu et al., 2020; Zhang et al., 2019) with different variations of limiting forwarding or, more generally, visibility. Furthermore, future research could analyze the differences between text-based and audio, photo, and video material, given that these materials have higher symbolic power, making disinformation more powerful (Durani et al., 2024).

### Contributions to practice

Our findings have implications for regulators, platform owners, and society. Regarding regulators, we conclude that privacy restrictions are insufficient to limit the harm of disinformation campaigns. Limiting what can be posted is difficult to justify and may be subject to claims of censorship (Helmore, 2020; Nuñez, 2019; Thompson & Hsu, 2024). Regulators may now have tools that limit the harm of disinformation campaigns simply by requiring that platforms restrict the reposting of the most suspect content. In the West, we have all been granted freedom of speech and expression by our constitutions and, in the case of the EU, by the EU Charter. However, these rights may not necessarily extend to unlimited amplification by online platforms with access to enough information to craft effective disinformation and target it with precision (Nuñez, 2019; Ressa, 2022; Wang et al., 2022). The restrictions we propose on limiting the reposting of the most harmful false content do not restrict what individuals can say. Still, they restrict how false information propagates and is amplified and algorithmically targeted. Platform operators may not wish to cooperate since disinformation leads to engagement, and engagement leads to profits. We expect the open-ended liability associated with risk-based compliance will lead to cooperation.

Platform operators need to be concerned about their legal liability for harm caused by disinformation, even if their platforms’ actions do not violate any explicit regulations: the presence of harm may be sufficient to demonstrate failure to implement adequate risk-based compliance. Platform operators can anticipate that disinformation campaigns can cause real societal and economic harm. Platform operators can anticipate that failure to limit the harm of disinformation can be seen as a failure of risk-based compliance and anticipate that they will experience real and perhaps significant economic penalties. This should cause platform operators to review the content they publish far more carefully than they do now. Even if platform operators are reluctant to restrict what can be posted, to avoid the appearance of censorship, they may be required to limit the reposting of the most inflammatory and suspect stories from the most suspect sources to limit their liability.

The societal implications are straightforward. Disinformation is harmful and results in societal polarization, increased xenophobia, and increased acts of violence from local internal actors. As important, even if less dramatic, is the fact that disinformation undermines democracy, leads to less informed or more misinformed electorates, and reduces individuals’ ability to act in their own best interest; consequently, disinformation represents an assault on human dignity and thus represents a violation of Article 1 of the EU Charter of Fundamental Human Rights (European Union, 2000). The relatively straightforward action of reducing forwarding and reposting of the most egregious disinformation is a promising mechanism for restoring this fundamental right.

## Conclusions, limitations, and future research

Our Face Validity Treatments (Table 2) allowed us to assess the factors contributing to disinformation campaigns’ effectiveness. Backlash is shown to be critical; when campaigns are observed by individuals not sympathetic to the campaign, these individuals become more committed in their opposition, more convinced of their initial beliefs, and more passionate in their defense of those beliefs. Since backlash is critical in determining the effectiveness of disinformation campaigns, reducing backlash is likewise critical to the effectiveness of these campaigns. This, in turn, means that better crafting of stories to appeal to segments of readers is important, and better targeting of those stories is important; these lead to less leakage of individual stories and shorter campaigns with less leakage overall.

Our Data Sharing Treatments allowed us to assess what disinformation campaigns can still accomplish, even with strict enforcement of GDPR and privacy controls. Data sharing between the platform and the disinformation campaign is a powerful enabler of the campaign, but we showed that aggregate data on the beliefs of segments of users is sufficient for the crafting of effective disinformation campaigns if the social media platform actively directs stories to users most likely to be sympathetic to them and influenced by them.

Finally, our Forwarding Treatments examined the most effective regulatory responses available through restrictions that can be enforced algorithmically without the appearance of censorship or bias. Regulators’ best hope of limiting the spread of disinformation without violating concerns about freedom of speech and censorship comes from reducing the forwarding of disinformation when this is based on objective assessments of the stories themselves and their sources.

Our research is subject to all the limitations of simulation research. Our results indicate the direction and relative impacts of different factors but are not and cannot be calibrated to specific disinformation campaigns within specific elections based on a detailed understanding of voters, their preferences, and their responses to individual stories. We cannot even say that accelerating backlash by two periods will or will not have a significant impact or that backlash is 50%, 75%, or 90%, equivalent to the impact of stories on their intended readers. We identify critical parameters and the nature of their interactions and perform sensitivity analysis to assess the stability of our results and to examine how small changes in parameter values affect outcomes.

We need to review our recommendations with regulators in the EU and the partisan US Congress to see if any regulation that limits disinformation will appear to censor one party or another. Moreover, this work can never be considered finished. We must understand that the battle between disinformation offense and defense will continue evolving as technology changes. Chatbots and predictive text generation techniques (Sebastian, 2023) will require constant adjustment to algorithmic protections. AI makes the crafting of disinformation faster and easier, and deepfakes make disinformation campaigns more challenging to detect and, thus, more effective. Increases in technological capability make social media disinformation campaigns more powerful. As reported in the press, disinformation contributes to societal polarization, xenophobia, racism, and the breakdown of multinational organizations. Consider the social media platforms to the rise of ethnic violence, as seen in Myanmar (Amnesty International, 2022; De Guzman, 2022). Consider the role of social media in Brexit (Cadwalladr, 2017; Kaiser, 2019) and the reelection of President Trump (Duffy, 2024). If mechanisms to limit the harm of these platforms do not advance to counter the increased power of their disinformation campaigns and their growing threat to society, they can evolve into an existential threat to human civilization.

For future research, we will work with our law faculty colleagues to assess the feasibility of algorithmic intervention and ensure that this does not cross over into censorship or partisan politics. Our simulation model will continue to evolve as the actions of disinformation campaigns, social media platform operators, and regulators evolve, requiring updates to the model.

## Supplementary Information

Below is the link to the electronic supplementary material.

Supplementary file1 (DOCX 32 KB)

## Footnotes

1 We thank one of the anonymous referees for highlighting that disinformation may contain both false and true information.

2 We thereby build on the baseline model described in Clemons (2018) on the spread of fake news and enhance it by including interventions to limit the spread. The original code for the baseline model used in Clemons (2018) was available to us.

3 This subject is complex and is dynamic and changing rapidly. Facebook originally argued that it was permitted to use personal data on its customers, internally with Facebook, to target ads and content to individual customers. The argument was that this was permitted because the use of data was permitted to improve the user experience, and could even be considered essential to delivering quality service as required by their contractual obligations to their users (Meta, 2023). The EU has subsequently determined that the use of personal data to target ads to specific users is not permitted (Fung, 2023), which could effectively limit or even eliminate paid disinformation campaigns. However, two questions still remain unresolved. (1) Is Facebook permitted to target false content to users who will respond favorably to it if it is not paid content, posted by true believers or by operatives paid to post the content, much as they might be paid to post fake product reviews on Amazon? (2) Is Facebook permitted to target reposted false content to users who will respond favorably to it, regardless of the source of the original post? (3) In particular, is Facebook allowed to target reposted content that was, itself, originally a paid ad that was part of a disinformation campaign?
