# Supplementary material for: Managing disinformation on social media platforms
Source: Electron Mark. 2025 Jun 9;35(1):52. doi: 10.1007/s12525-025-00796-6 (PMC12149016; doi:10.1007/s12525-025-00796-6)
Supplement: Supplementary file 1 — Supplementary file1 (DOCX 32 KB) [file 12525_2025_796_MOESM1_ESM.docx]

# Appendix

## Appendix A—Construction of the Model

The most important part of constructing a simulation model is determining which parameters need to be included and how they interact. Some of these parameters may be subject to the modeler’s control, the decision variables. Other parameters may be determined by the state of the world being modeled and are not subject to the modeler's control. For these parameters, the modeler may have access to historical data, while for others, no data is available; when no historical data values are available, the modeler must use intuition to set initial values and should do sensitivity analysis, varying these parameters, to determine how sensitive the model is to changes to these initial values. Below is a brief overview of the steps we followed when constructing the simulation. We thereby build on the baseline model described in Clemons (2018) on the spread of fake news and enhance it by including interventions to limit the spread.

1. We set our overall goal for the simulation. We want to understand how the support for Red and Green changes over time during a disinformation campaign conducted by Red, so we need to measure support for Red and Green at each point in time.
2. We set how voters will behave when the actual election is held. We modelled that voting depended upon agreement with Red and Green’s position (proximity to it) and on the intensity of the voter’s commitment. That is, we modeled changes to both *what* the voter believed and *how strongly* the voter believed it.
3. We set the initial conditions, which in this case are represented by the initial levels of support for Red and Green. We wanted to start with Red having the more extreme position and with Red having less support. We started with Red having less support because that way we could see when a disinformation campaign succeeded in changing the outcome. We also started with Red occupying a more extreme position because data suggests that extreme positions are more likely to deploy disinformation campaigns and are more likely to be influenced by them (Bradshaw et al., 2020)
4. We defined how stories would be created and how they would be directed to Red and Green voters.

- We allowed stories to be targeted or not. A targeted story went only to voters sympathetic to Red, while an untargeted story went to all voters.
- We allowed stories to be precision-crafted or not. If stories were not precision-crafted, the same story went out to all voters or all Red voters, depending on whether stories were targeted. If stories were precision-crafted, then there were groups of stories at each point in a campaign, and within each group, a story went only to those Red voters for whom it would be most effective.
- Finally, we had to consider leakage, that is, whether stories directed to Red voters might eventually reach Green voters, and if so, when.

1. Fifth, we had to determine the impact of a disinformation campaign on Red and Green voters

- Disinformation stories read by voters sympathetic to Red make some readers more sympathetic to Red (move their location in voter space), some voters more committed in their beliefs, or both.
- Disinformation stories read by voters sympathetic to Green make some readers more hostile to Red (move their location in voter space), some voters more committed in their beliefs, or both.
- We assumed that backlash among Green voters was less powerful than the direct impact of the stories on Red voters, and showed through sensitivity analysis that the difference in impact could determine whether or not a disinformation campaign succeeded.

1. Next, we related these phenomena to information sharing by the platform:

- If the platform shares information on individual voters with Red’s disinformation campaign, this allows better targeting, which reduces Green sympathizers receiving Red’s disinformation campaign, and thus reduces backlash.
- If the platform shares aggregate information with Red’s disinformation campaign, this allows for more precise crafting of stories for each segment of Red voters. Combined with information on individual voters, this also allows better targeting. More precise crafting allows shorter disinformation campaigns, which again reduces Green sympathizers receiving Red’s disinformation campaign, and thus reduces backlash.

1. Next, we set reasonable initial values for parameters.

- This did not mean setting the parameters to actual values observed in practice because these would differ across all disinformation campaigns and perhaps also across stories within a disinformation campaign.
- Rather, we set parameters on how much stories affected Red and Green sympathizers to demonstrate that the model could produce interesting results. That is, we chose parameter values such that Red did not succeed without some targeting and did succeed with targeting. We did a sensitivity analysis to demonstrate that there were settings for which Red’s victory did succeed without targeting and that Red’s victory did not succeed even with targeting.
- We did a sensitivity analysis to explore how the timing of leakage affected Red’s success. If the leakage was early, then the backlash blocked Red’s victory. If leakage was gradual, with some leakage occurring early and some occurring late, then the timing and speed of the leakage mattered.

1. We next explored how information sharing between the platform and the disinformation campaign improved the effectiveness of the disinformation campaign. We did this by assuming that shared information allowed better crafting of stories, better targeting of stories, or both. Better crafting of stories increased the impact of disinformation on Red sympathizers, while the backlash remained unchanged. Better targeting reduced backlash, while the impact on Red sympathizers remained unchanged. To implement this, we altered the parameters that define the impact of disinformation on Red sympathizers and the impact of backlash. The resulting impact on Red and Green votes is reported in Table 3 in the main document.
2. Lastly, we explored the impact of forwarding restrictions. As described in the main document, we introduce additional parameters to model the spread of disinformation through forwarding by influencers to susceptible readers, either at once or in waves. Specifically, this increased the impact of disinformation on Red sympathizers, but the number of Red sympathizers who see the disinformation content is controlled by the forwarding scheme modeled in the simulation.

Overall, we did not attempt to do any comparisons with data from specific campaigns. Each campaign would have a different distribution of voters and different parameters for influence from stories or for backlash for stories. We are not attempting to develop a regulatory policy for France (EU), the UK (non-EU European democracy), or the US (non-European democracy). We are attempting to show how our structural model responds to parameter changes.

## Appendix B—Parameters of the Simulation and Their Values

| **Parameter** | **Values** | **Reasoning** |
| --- | --- | --- |
| *Impact of targeted disinformation on sympathetic voters (adjacent segments in the voter-attitude space)***^†^** | | |
| Percentage of voters’ attitude change | 16 % | - Conservative assumptions based on Nisbet et al. (2021) - Relative advantage compared to backlash varied in sensitivity analysis |
| Percentage of voters’ commitment change | 12 % |  |
| Percentage of voters with both attitude and commitment change | 9 % |  |
| *Impact of disinformation on unsympathetic voters (backlash)***^††^** | | |
| Percentage of voters’ attitude change | 12 % | - Disinformation campaigns can draw backlash (Weir, 2024) - The impact of backlash is lower than the impact of the disinformation campaign on sympathetic readers |
| Percentage of voters’ commitment change | 8 % |  |
| Percentage of voters with both attitude and commitment change | 6 % |  |
| Backlash Starts | 0; 2; 5; 8 (period) | Targeting disinformation campaigns will delay the start of backlash because stories will first spread only within filter bubbles (Arguedas et al., 2022) |
| Backlash Intensity | 0.25; 0.65; 0.75 | The backlash among voters sympathetic to Green might vary across groups and communities (cf. Weir, 2024) |
| Backlash Ramp-Up Length | 10; 20 (periods) | Backlash might not occur at once, but ramp up over a certain number of periods (cf. Weir, 2024) |
| Red Precision Increase | 0; 0.2; 0.25 | Targeted disinformation has more impact than untargeted disinformation (Woolley & Howard, 2018) |
| Campaign Length | 8; 15; 36; 75 (periods) | Election and disinformation campaigns play out over time (Stevenson & Vavreck, 2000; Lukito, 2020) |
| Influencers | 50; 100 | Influencers play an increasing role in disseminating content related to elections (Goodwin et al., 2023) |
| Influencer Impact | 4 | Influencers have persuasive power over consumers (Liu & Zheng, 2024), and we assume that this translates to the political sphere as well |
| Forwards | 6; 9; 10; 1,000 | - Disinformation spreads through mechanisms such as linking, commenting, and sharing of posts (Ng et al., 2021) - Forwarding occurs in waves as disinformation is being shared |
| Waves | 1; 3 |  |

Table B.1. Summary of model parameter values

**Notes: ^†^** This set of parameters determines the impact of each disinformation story on the beliefs and on the intensity of beliefs of the intended population; i.e., the population that is sympathetic to Red’s position. The parameters determine how much each disinformation story benefits Red. If the parameters were higher, successful disinformation campaigns could be shorter, and if they were lower, successful disinformation campaigns would need to be longer. We chose the values we did because the resulting campaign lengths allowed us to explore dynamic behavior with runs of reasonable length. We are exploring the shape of possible trajectories and not modeling any specific campaign in any specific election.

**^††^**This set of parameters determines the impact of each disinformation story on the beliefs and on the intensity of beliefs of the other population; i.e., the population that is unsympathetic to Red’s position. The parameter determines how much each disinformation campaign can weaken Red’s position. The lower the parameters are, the less backlash will occur, and if the parameters are zero, no targeting is necessary, and disinformation campaigns will always succeed, given our other parameter settings. Alternatively, suppose the parameters are equal to those that determine each disinformation campaign's impact on Red’s supporters. In that case, the balance between Red and Green will never change, and no disinformation campaign can succeed, no matter how long it runs.

# References

Arguedas, A. R., Robertson, C. T., Fletcher, R., & Nielsen, R. K. (2022). *Echo Chambers, Filter Bubbles, and Polarisation: a Literature Review*.

Bradshaw, S., Bailey, H., & Howard, P. N. (2020). *Industrialized Disinformation: 2020 Global Inventory of Organized Social Media Manipulation*.

Clemons, E. K. (2018, October 11). How Private Information Helps Fake News Fool the Public. *Knowledge@Wharton*. <https://knowledge.wharton.upenn.edu/article/how-private-information-helps-fake-news-to-hoodwink-the-public/>

Goodwin, A., Joseff, K., Riedl, M. J., Lukito, J., & Woolley, S. (2023). Political relational influencers: The mobilization of social media influencers in the political arena. *International Journal of Communication*, *17*, 21.

Liu, X., & Zheng, X. (2024). The persuasive power of social media influencers in brand credibility and purchase intention. *Humanities and Social Sciences Communications*, *11*(1), 15.

Lukito, J. (2020). Coordinating a multi-platform disinformation campaign: Internet Research Agency Activity on three US Social Media Platforms, 2015 to 2017. *Political Communication*, *37*(2), 238-255.

Ng, K. C., Tang, J., & Lee, D. (2021). The Effect of Platform Intervention Policies on Fake News Dissemination and Survival: An Empirical Examination. *Journal of Management Information Systems*, *38*(4), 898-930.

Nisbet, E. C., Mortenson, C., & Li, Q. (2021). The presumed influence of election misinformation on others reduces our own satisfaction with democracy. *Harvard Kennedy School Misinformation Review*, *1*.

Stevenson, R. T., & Vavreck, L. (2000). Does campaign length matter? Testing for cross-national effects. *British Journal of Political Science*, *30*(2), 217-235.

Weir, K. (2024). This election year, fighting misinformation is messier and more important than ever. *Monitor on Psychology*, *55*(1), 40.

Woolley, S. C., & Howard, P. N. (2018). *Computational Propaganda: Political Parties, Politicians, and Political Manipulation on Social Media* (S. C. Woolley & P. N. Howard, Eds.). Oxford University Press.
